# Factors associated with the use of outpatient services among the elderly in Indonesia

**DOI:** 10.1186/s12913-018-3512-0

**Published:** 2018-09-11

**Authors:** Ema Madyaningrum, Ying-Chih Chuang, Kun-Yang Chuang

**Affiliations:** 1 School of Public Health, Taipei Medical University, 250 Wu-Hsing Street, Taipei, 11014 Taiwan; 2 School of Nursing, Faculty of Medicine, Universitas Gadjah Mada, Farmako Street, Sekip Utara, Yogyakarta, 55281 Indonesia

**Keywords:** Outpatient services, Elderly, Indonesia, Health care, Utilization

## Abstract

**Background:**

Elderly people tend to have greater healthcare utilization because of their health status. However, with the 4th largest population in the world, little is known concerning the use of services among the Indonesian elderly. Hence, this study aimed to identify factors related to the use of outpatient services among the Indonesian elderly.

**Methods:**

This is cross sectional study using data from the Indonesian Family Life Survey 5 (IFLS 5), conducted in 2014 and 2015. Only those who were 60 years or older were included in the analyses. We used a logistic regression analysis to determine factors associated with use of outpatient services.

**Results:**

Among 2912 participants, only 22.7% of respondents had visited health workers or doctors within the previous 4 weeks before the survey. After controlling for other variables, factors associated with the use of outpatient services were socioeconomic status, insurance status, regions of residence, self-rated health, and the number of chronic conditions.

**Conclusion:**

Inequality in outpatient services was observed among the elderly in Indonesia. More effort is need to expand insurance coverage for the elderly, particularly for those in the lower economic status, and to improve access to outpatient services in rural regions by allocating the needed resources.

## Background

The United Nations estimates that the number of people aged ≥60 years will increase from 901 million in 2015 to 1.4 billion in 2030 globally, an increase of over 56% in 15 years. It was estimated that 71% of the increase in the elderly population will occur in developing countries [1]. Indonesia, with the 4th largest population in the world of around 258 million in 2015, is also facing challenges of a rapidly increasing elderly population. The number of elderly increased by about 4.12 million from 2000 to 2015, as the life expectancy in Indonesia increased from 67.25 to 70.8 years in the same period [2]. With an increase in life expectancy and a reduction in fertility, it is expected that the rate at which the population ages will also increase. By the year 2025, it is estimated that 11.8% of the population will be ≥60 years [3].

Previous research indicated that the elderly tend to have a lower self-rated health status [4], more instances of comorbidities [5, 6], and polypharmacy [7], and these factors are associated with an increased use of outpatient services [8, 9]. Hence, as the elderly population increases, demands for outpatient services will also likely increase [10]. While one’s health status is an important determinant of outpatient visits, sociodemographic and cultural factors, financing, and the availability of regional resources may also influence utilization of outpatient visits.

Currently, in Indonesia, outpatient care services are mostly provided by doctors in public hospitals, Puskesmas (public health centers), private hospitals, and private clinics. In rural areas, however, nurses and paramedics also provide outpatient care due to a shortage of doctors. The ability to use outpatient services had a significant influence on access to other publicly funded health care services [11]. In the Indonesian health care system, primary outpatient services acted as the gatekeeper to services, such as specialist visits at the provincial hospital, or inpatient hospitalization. These services would not be covered without referral from the primary outpatient care providers.

Indonesia is a country with 34 provinces spread over more than 17,000 islands. Resources and culture tend to vary from province to province. Ensuring a sufficient and equitable distribution of healthcare resources for its increasing elderly population has become a challenging task. At present, little is known about the utilization of outpatient care and barriers to care among the Indonesian elderly. Previous studies identified multiple comorbidities, self-rated health, and socioeconomic status as factors related to the use of outpatient services in developing countries [9, 12, 13]. Hence, this study aimed to identify factors related to the use of outpatient services among the Indonesian elderly. Results of this research may help to identify possible inequality in the use of outpatient services. Although the national health insurance in Indonesia has been launched in 2014, it has been a challenge to achieve universal coverage. Elderly in the urban areas were more likely to be enrolled in the national health insurance than those in the rural area, and disparity in enrollment were observed among provinces [14]. Overall, this research can provide some preliminary evidence on the extent of health care coverage among the elderly in Indonesia, as well as inequality in use of outpatient services. The findings from this research may contribute to the formulation of elderly healthcare policies, particularly in the resource allocation for the deprived areas, and access to care for the underprivileged elderly.

## Methods

### Study design

This was a cross-sectional study, using data obtained from the Indonesian Family Life Survey (IFLS), conducted in 2014 and 2015. Briefly, the IFLS was conducted by the RAND Corporation in collaboration with Lembaga Demografi, Universitas Indonesia, University of California, Los Angeles (UCLA) in the USA, and the Center for Population and Policy Studies of The Universitas Gadjah Mada. The IFLS was first conducted in 1993, and was carried out in 1997, 2000, 2007, and 2014. The IFLS aims to provide an overview of behaviors and health outcomes of the general population in Indonesia, and hence, contains information about the use of health services, physical health, mental health, health behaviors, and basic sociodemographic characteristics including household assets. A total of 13 provinces, representing 83% of the total population, participated in the survey [15]. Detailed information about the study design of the IFLS can be obtained from http://www.rand.org/labor/FLS/IFLS.html.

For this research, data from elderly respondents in IFLS 5 was used. An elderly was defined as someone who was 60 or older, when the person was eligible to receive old-age social welfare benefits in Indonesia. The criterion was based on the guideline issued by The Government of Indonesia [16]. The number of elderly people who participated in IFLS 5 was 4007 people. After excluding proxy respondents and those respondents with missing data, 2912 respondents were included for analysis.

### Measurements

Five questions were used to measure the use of outpatient care. The elderly were asked whether they had had an outpatient visit with a doctor or health worker in either a (1) public hospital, (2) a private hospital, (3) a Puskesmas (public health center), (4) a doctor’s office, or (5) a paramedic or nursing clinic in the past 4 weeks at the time of interview. If an elderly person had used any of the services, they were coded as having used outpatient services.

Based on Andersen’s model of health service utilization, need, enabling, and predisposing factors were selected as predictors of outpatient services [17]. For this research, need factors included self-rated health, chronic conditions, depression, functional status, prior incidence of a fall, and hospitalization within the last year. The question used to ascertain self-rated health was *“In general, how is your health?”*, and the response categories were “very healthy”, “somewhat healthy”, “somewhat unhealthy”, and “unhealthy”. The number of chronic conditions was tabulated based on the following 12 chronic conditions: hypertension, diabetes or high blood sugar, tuberculosis, asthma, heart attack or other heart problems, liver diseases, stroke, cancer or malignant tumors, arthritis, high cholesterol, kidney diseases, and digestive diseases. Respondents were asked whether they had a specific condition, and whether it had ever been diagnosed by a doctor. Depression was measured with the Center of Epidemiological Studies Depression Scale-10 (CESD-10). The questions were: *I was bothered by things that usually don’t bother me; I had trouble concentrating on what I was doing; I felt depressed; I felt everything I did was an effort; I felt hopeful about the future; I felt fearful; My sleep was restless; I was happy; I felt lonely;* and *I could not get going.* These 10 items were coded in the same direction from 0 to 3 and were added. The total score ranges 0~ 30, with a score of > 10 indicating possible depression [18]. One’s functional status was assessed by five measures of activities of daily living (ADLs), including getting dressed, bathing, getting out of bed, eating, and toileting. Those who had difficulty independently completing any of the five activities were deemed as having functional limitations.

The question used to measure whether the elderly had experienced a prior incidence of fall was “*Have you fallen down in the last 2 years?”* For hospitalization within the past year, the question was “*During the past 12 months, have you ever received inpatient care at a hospital,* Puskesmas*, clinic, or other*?”

Enabling factors mainly dealt with whether the person had access to health services. In this study, household assets, the insurance status, residence, main language, and island were used to measure the financial, cultural, and geographical barriers to seeking care. In IFLS, respondents were asked about the value of their house, land, livestock and vegetation of economic value, vehicles, household appliances, household furniture, savings or deposits, receivables, jewelry, and other assets. The total value of various assets was used to represent economic status, and respondents were divided into quartiles. Several types of insurance are available for the elderly in Indonesia, including Asuransi Kesehatan (ASKES), Asuransi Tenaga Kerja (ASTEK) / Jaminan Sosial Tenaga Kerja (JAMSOSTEK) for labor insurance, employer-provided health insurance, savings-related insurance, Jaminan Kesehatan Masyarakat (JAMKESMAS), Jaminan Kesehatan Daerah (JAMKESDA), Jaminan Kesehatan Nasional (JKN), and private insurance. Respondents were asked if they had any of those health insurance programs. Residence location was categorized as either “rural” or “urban” based on the Indonesian Central Bureau of Statistics (BPS) code. Language represents the capability of elderly people to communicate and access health services. The official language is the Indonesian language. Another commonly used language is Javanese. Language was divided into “Indonesian”, “Javanese”, and “other”. In this study, the regional variable used the major island category as its basis of location. We categorized the region variable into “Java”, “Sumatera”, “Kalimantan”, “Sulawesi”, “Bali”, and “West Nusa Tenggara” regions.

Predisposing characteristics included age, gender, marital status, education, and religion, which were covariates. Age categories were “60~ 64”, “65~ 69” “70~ 74”, and “≥75” years. Marital status categories were “married”, “widowed”, and “other” which included single and divorced respondents. Respondents were asked whether they had ever attended any school, and if so, the type of school. The educational level was then divided into “no formal education”, “elementary school”, and “junior high school or higher”. Since over 85% of the population in Indonesia were Muslims, religion variable was coded as “Muslim” and “others”.

### Statistical analysis

Basic characteristics of participants were presented with numbers and percentages, and bivariate analysis of respondent characteristics and the use of outpatient services was conducted with Pearson Chi-square test. A multivariate logistic regression analysis was performed to determine factors associated with outpatient care utilization. A 95% confidence interval (CI) was used to report the percentage of the odds ratio (OR). All analyses were performed using SPSS for Windows version. 19 (SPSS, Chicago, IL, USA).

## Results

Table 1 shows the basic characteristic of the 2912 participants. Among them, 51.6% were females, and 42.3%, 24.8%, 19.5%, and 13.4% were respectively in the 60~ 64, 65~ 69, 70~ 74, and ≥ 75 year age groups. Most respondents had an elementary level education, while 19% had no formal education, and only 26.2% had an educational level equivalent to junior high school or higher. About 46.9% of the elderly people had insurance, slightly lower than previously reported in 2013 (51.93%) [19]. In this study, 54.3% of participants resided in urban areas, consistent with the government report that 54% of the total population lives in urban areas [14].

**Table 1:** Frequency distribution of characteristics among Indonesian elderly (*N* = 2912)

Variable	Frequency (n)	Percent (%)
Sex		
Male	1409	48.4
Female	1503	51.6
Age		
60~ 64	1232	42.3
65~ 69	722	24.8
70~ 74	568	19.5
≥ 75	390	13.4
Marital status		
Married	1888	64.8
Widowed	920	31.6
Other	104	3.6
Educational background		
No formal education	552	19.0
Elementary school	1596	54.8
Junior high school or higher	764	26.2
Religion		
Moslem	1543	87.3
Others	369	12.7
Economic status		
1st [lowest]	729	25.0
2nd	727	25.0
3rd	728	25.0
4th [highest]	728	25.0
With Insurance		
No	1547	53.1
Yes	1365	46.9
Residency		
Urban	1582	54.3
Rural	1330	45.7
Main language		
Indonesian	465	16.0
Javanese	1120	38,5
Other	1327	45,6
Region		
Java	1756	60.3
Sumatera	567	19.5
Kalimantan	124	4.3
Sulawesi	133	4.6
Bali	137	4.7
West Nusa Tenggara	195	6.7
Self-rated health		
Very healthy	364	12.5
Somewhat healthy	1471	50.5
Somewhat unhealthy	997	34.2
Unhealthy	80	2.7
Number of chronic conditions		
None	1783	61.2
1	633	21.7
2 or more	496	17.0
Depression		
No	2186	75.1
Yes	726	24.9
Difficulty with activities of daily living		
No	2494	85.6
Yes	418	14.4
Ever fallen down before		
No	2556	87.8
Yes	356	12.2
Hospitalized in the past year		
No	2758	94.7
Yes	154	5.3
Visited health workers or doctors in the past 4 weeks		
No	2250	77.3
Yes	662	22.7

In general, the data showed a generally healthy elderly population in Indonesia. More than 60% of the elderly rated their own health as “very healthy” or “somewhat healthy”, and only 2.7% as “unhealthy”. The self-rated health statistics are similar to those of a study conducted in Singapore, where 65.6% of the elderly rated their health as being good or better [20]. Compared to another study carried out in Java in 2010, the results are similar with 68.4% of the elderly rated as being in good health [21]. The percentage of elderly showing possible signs of depression was 24.9%, slightly higher than what was observed in 2007 [22]. In terms of ADL functions, 14.4% indicated that they had difficulty in at least one of the five ADL functions.

In terms of healthcare utilization, 5.3% of participants had been hospitalized in the past year, slightly lower than the 7.2% reported in the Indonesia Central Bureau of Statistics 2015 [14]. Overall, 22.7% of respondents had visited health workers or doctors within the previous 4 weeks before the survey.

Table 2 shows the results of bivariate analysis of respondent characteristics and the use of outpatient services. With the exception of age and marital status, all the other variables were significantly associated with the use of outpatient services. In general, being female, higher educated, higher economic status, worse health, and urban regions were associated with the use of outpatient services. Table 3 shows results of the multivariate logistic regression. Odds ratio and 95% confidence intervals are presented. After controlling for other variables, the economic status, health insurance, region, self-rated health, and the number of chronic conditions were statistically significantly associated with the use of outpatient services. Compared to those in the lowest quartile of the economic status, the 2nd, 3rd, and, 4th quartiles had respective OR; 1.36 (95% CI: 1.03–1.78), 1.32 (95% CI: 1.00–1.74), and 1.36 (95% CI: 1.02–1.81). Those with health insurance were 1.38 (95% CI: 1.14–1.67) times more likely than their counterparts to use outpatient services. Compared to the healthy elderly, those who rated themselves as somewhat unhealthy and very unhealthy were 2.1 (95% CI: 1.49–2.95), and 2.22 (95% CI: 1.24–3.96), times more likely to visit outpatient care. Having more chronic conditions was also associated with higher use of outpatient services. This model showed a good fit, and had an R-square of 0.139.

**Table 2:** Bivariate analysis of factors associated with outpatient visits among Indonesian elderly

Variables	Participants using outpatient services		Participants not using outpatient services	
	N	%	N	%
Sex**				
Male	291	20.7	1118	79.3
Female	371	24.7	1132	75.3
Age				
60~ 64	273	22.2	959	77.8
65~ 69	170	23.5	552	76.5
70~ 74	128	22.5	440	77.5
≥ 75	91	23.3	299	76.7
Marital status				
Married	429	22.7	1459	77.3
Widowed	216	23.5	704	76.5
Other	17	16.3	87	83.7
Educational background***				
No formal education	120	21.7	432	78.3
Elementary school	326	20.4	1270	79.6
Junior high school or higher	216	28.3	548	71.7
Religion**				
Moslem	555	21.8	1988	78.2
Others	107	29.0	262	71.0
Economic status ***				
1st [lowest]	127	17.4	602	82.6
2nd	167	23.0	560	77.0
3rd	166	22.8	562	77.2
4th [highest]	202	27.7	526	72.3
With Insurance***				
No	288	18.6	1259	81.4
Yes	374	27.4	991	72.6
Residency***				
Urban	397	25.1	1185	74.9
Rural	265	19.9	1065	80.1
Main language***				
Indonesian	129	27.7	336	72.3
Javanese	220	19.6	900	80.4
Other	313	23.6	1014	76.4
Region**				
Java	396	22.6	1360	77.4
Sumatera	122	21.5	445	78.5
Kalimantan	24	19.4	100	80.6
Sulawesi	30	22.6	103	77.4
Bali	51	37.2	86	62.8
West Nusa Tenggara	39	20.0	156	80.0
Self-rated health***				
Very healthy	51	14.0	313	86.0
Somewhat healthy	261	17.7	1210	82.3
Somewhat unhealthy	322	32.3	675	67.7
Unhealthy	28	35.0	52	65.0
Number of chronic conditions***				
None	268	15.0	1515	85.0
1	183	28.9	450	71.1
2 or more	211	42.5	285	57.5
Depression***				
No	461	21.1	1725	78.9
Yes	201	27.7	525	72.3
Difficulty with activities of daily living ***				
No	537	21.5	1957	78.5
Yes	125	29.9	293	70.1
Ever fallen down before**				
No	560	21.9	1996	78.1
Yes	102	28.7	254	71.3
Hospitalized in the past year***				
No	607	22.0	2151	78.0
Yes	55	35.7	99	64.3

Note: ** *p* < 0.01; *** *p* < 0.001

**Table 3:** Factors associated with outpatient visits among Indonesian elderly

Variable	Odds ratio	95% Confidence interval
Sex		
Male	1.00	–
Female	1.19	0.96–1.48
Age		
60~ 64	1.00	–
65~ 69	1.09	0.86–1.37
70~ 74	1.02	0.79–1.32
≥ 75	1.19	0.88–1.61
Marital status		
Married	1.00	–
Widowed	0.96	0.76–1.20
Other	0.65	0.37–1.14
Educational background		
No formal education	1.00	–
Elementary school	0.80	0.76–1.20
Junior high school or higher	0.99	0.71–1.39
Religion		
Moslem	1.00	–
Others	1.01	0.73–1.39
Economic status		
1st [lowest]	1.00	–
2nd	1.36*	1.03–1.78
3rd	1.32*	1.00–1.74
4th [highest]	1.36*	1.02–1.81
With Insurance		
No	1.00	–
Yes	1.38**	1.14–1.67
Residency		
Urban	1.00	–
Rural	1.01	0.82–1.24
Main language		
Indonesian	1.00	–
Javanese	0.92	0.69–1.22
Other	0.99	0.74–1.33
Region		
Java	1.00	–
Sumatera	0.85	0.65–1.11
Kalimantan	0.82	0.50–1.35
Sulawesi	0.82	0.51–1.33
Bali	1.82*	1.10–3.01
West Nusa Tenggara	0.85	0.56–1.30
Self-rated health		
Very healthy	1.00	–
Somewhat healthy	1.13	0.81–1.57
Somewhat unhealthy	2.10***	1.49–2.95
Very unhealthy	2.22**	1.24–3.96
Number of chronic conditions		
None	1.00	–
1	1.87***	1.49–2.35
2 or more	3.01***	2.36–3.85
Depression		
No	1.00	–
Yes	1.13	0.91–1.40
Difficulty with activities of daily living		
No	1.00	–
Yes	1.24	0.96–1.60
Ever fallen down before		
No	1.00	–
Yes	1.11	0.85–1.46
Hospitalized in the past year		
No	1.00	–
Yes	1.24	0.86–1.79

Note: * *p* < 0.05; ** *p* < 0.01; *** *p* < 0.001. Hosmer and Lemeshow Test of goodness of fit: *p* = 0.443 (>.05); Nagelkerke R^2^ = 0.139

## Discussion

This is the first study to report on factors associated with the use of outpatient services among the elderly in Indonesia. In this study, nearly 23% of the elderly had had at least one outpatient visit within the last 4 weeks from the time of the survey. Factors, such as health insurance, economic status, region, self-rated health, and chronic conditions, were significantly associated with outpatient utilization.

One’s insurance status was associated with higher use of outpatient services among the elderly probably due to removing financial barriers to care. For those elderly who are generally retired without a stable source of income, the availability of health insurance could have a significant impact on the use of health services, as well as on their health status. Under the current health system in Indonesia, the elderly may be covered under ASKES, JAMKESMAS, JAMKESDA, JAMSOSTEK, or private insurance. Briefly, ASKES is an insurance program for civil servant pensioners. JAMKESMAS and JAMKESDA are insurance programs for those below the poverty line. JAMKESMAS is funded by the central government, while JAMKESDA is funded by provincial or district governments. JAMKESDA is meant to be complementary coverage for those who are not covered by JAMKESMAS. JAMSOSTEK is social insurance that targets private sector employees. In 2014, all of these insurance schemes were integrated into a National Health Insurance, coordinated by Badan Penyelenggara Jaminan Sosial (BPJS) [23], and it was estimated that the proportion of the elderly with insurance had reached 54.58% in 2015 [14]. Despite the recent increase in coverage, nearly half of the elderly (46.9%) in this study were still without insurance.

Health insurance, in addition to increasing access to outpatient services, appeared to moderate the influence of economic factors in this research. Further analysis indicated (not shown here) that when stratified by the insurance status, the association between one’s economic status and the use of services became non-significant among those with insurance. The lack of insurance may decrease access to services, and likely lead to unmet needs and negative health outcomes among the elderly, particularly the poor elderly. It was estimated that almost half (45.14%) of the elderly were living below the poverty line in 2015 [14]. Although the Ministry of Health has several programs to encourage health promotion and prevention in villages by health volunteers, and facilitates collaboration between local governments and the Corporate Social Responsibility (CSR) to provide healthcare services for poor elderly people, these are probably insufficient. At present, no research has focused on the unmet needs of outpatient visits among the elderly in Indonesia. Further research could assess the relationship between unmet needs and the lack of insurance among the Indonesian elderly, and initiatives to increase insurance coverage of the elderly should be among the top policy priorities for improving accessibility of outpatient care.

Results also revealed that the use of outpatient services varied among regions. In this study, elderly persons living in Kalimantan and Sulawesi were less likely to use outpatient care compared to elderly persons in Bali. Such disparities were probably due to differences in healthcare coverage, in the availability of service providers. Previous research also identified that elderly persons in Bali were more likely to be covered under a health insurance program [24]. Greater physician resources, coupled with a higher percentage of the elderly with insurance, probably contributed to the higher utilization in Bali. Regional differences in resources were also apparent. In 2013, the number of physicians per 10,000 population was 7.04 in Bali, 2.47 in Kalimantan, and 3.08 in Sulawesi. In addition to health care manpower, physical infrastructure was also disproportionally distributed, with nearly half of the hospitals located in Java and Bali [25]. Over the years, the Indonesian government has taken initiatives to reduce geographical disparities in resources, such as providing financial incentives for healthcare workers to serve in remote, underserved areas. The program has had some success [26], but nevertheless, disparities remained. There have been efforts to increase the number of primary health care clinics (Puskesmas) in rural provinces, as well. The ratio between Puskesmas and population has reached 1:11,540 in rural provinces like Maluku, East Nusa Tenggara, West Nusa Tenggara, and Papua, even higher than those in Java and Bali (1:38,055). Nevertheless, access to Puskesmas remained difficult due to geographical obstacles [25]. To reduce geographical obstacles, one approach that can be considered is the use of mobile clinic that can bring basic primary care to remote regions. Approaches like utilizing telemedicine to increase access to primary or specialist services can also be considered if technology becomes available. Overall, Indonesia has one of the lowest physician-to-population ratios among nations in the Association of Southeast Asian Nations. It was estimated that the number of physicians per 10,000 population was about 19 for Singapore, 12 for Malaysia, and only 2 for Indonesia [27]. As the population ages, given the already low number of physicians, it will likely create more stress on an already strained supply of physicians and healthcare workers. Hence, to meet anticipated increases in demand for outpatient services, the Indonesian government should assess its current deficit in human resources in health care, and accordingly increase its investment in its educational system to develop appropriate numbers of healthcare providers.

Consistent with previous studies, the elderly with a worse self-rated health status and a higher number of chronic conditions were more likely to use outpatient services. In this research, nearly 36.9% of the elderly rated their health as somewhat unhealthy or unhealthy. This rate is comparable to those in neighboring countries of Singapore (34.4%) and Malaysia (39.9%), but slightly lower than what was observed in Korea (40.2%) [8, 20, 28–30]. The presence of chronic conditions, usually associated with decreased body function, was one of the most common predictor of healthcare utilization among the elderly. In this study, 38.7% of the elderly reported having at least one chronic condition, and nearly 20.3% of the elderly reported having hypertension, the highest level among chronic conditions. Such a finding is consistent with previous data reported in Indonesia. Similar results were reported in Malaysia and Korea as well [8, 31]. To deal with the increasing prevalence of hypertension and other chronic conditions, the Indonesian government initiated programs to improve the health status of the elderly, such as health lifestyle programs (with a focus on a healthy diet and physical activities), early detection and counseling programs in primary health care, and community empowerment programs by POSYANDU LANSIA (Integrated Health Post) that provide preventive and health promotion by local communities.

### Strengths and limitations

This is the first study to explore factors associated with outpatient care utilization among the elderly in Indonesia, and has identified factors that were significantly associated with outpatient utilization. Nevertheless, it has several limitations. Only those elderly who were able to respond to the questionnaire and without missing data were included in the analysis, hence the generalizability of research finding ought to be interpreted accordingly. Elderly who were bedridden, institutionalized, or unable to communicate were excluded from the analysis, and hence the very low response rate. Those excluded from the analysis were older, having less education, and more likely to be females than those included in the analysis, and hence, it is likely that the need for outpatient services might be underestimated. Moreover, those excluded from analyses may have access problem to outpatient services. For those excluded from the analysis, particularly those institutionalized or bedridden, the relationship between the need for, access to service, and eventually the actual use of service is an area worth further research. More research is needed to understand factors associated with outpatient services, in particular how the role of caregiver may have influences their access to services. In the analysis, the use of regions did not represent actual provinces, but was simplified based on islands. Even though provinces on the same island likely have similar culture and resources, provinces still slightly differ in health resources. Finally, the cross-sectional study design cannot explicitly imply causality, even though the direction of the influence is consistent with previous research.

## Conclusions

The need for outpatient care is likely to increase as the population ages in Indonesia. In addition to need factors, such as self-rated health and chronic conditions, the other main factors associated with the use of outpatient services among Indonesian elderly were socioeconomic status, having insurance, and regions. The significant influence of socioeconomic status, insurance status, and the region indicated possible signs of healthcare inequality issues that needed policy intervention. Overall, ensure accessibility to outpatient services for the elderly will likely require significant inputs of resources and administrative efforts from regional and central governments. Despite of the recently implemented national health insurance, more than half of the elderly remain without insurance coverage, and hence limited access to outpatient services. The government should continue to scale up efforts to increase coverage rate for the elderly, especially for the economically disadvantaged. Regional difference in outpatient utilization should be ameliorated by increasing healthcare manpower in rural provinces, as well as considering other innovative models of care delivery, such as mobile outpatient clinic or telemedicine consultations, in order to reduce the impact of geographical barriers on outpatient utilization. Future studies on elderly use of services should also focus on exploring roles of family and community in health care utilization, including primary care visits, specialist care, and inpatient hospitalization services. The extent that the recently implemented national health insurance was able to moderate the effect of socioeconomic status on use of services should also be assessed.

## Abbreviations

ASKES: Asuransi Kesehatan
ASTEK: AsuransiTenaga Kerja
BPJS: Badan Penyelenggara Jaminan Sosial
CESD-10: Center of Epidemiological Studies Depression Scale-10
CSR: Corporate Social Responsibility
IFLS: Indonesia Family Life Survey
JAMKESDA: Jaminan Kesehatan Daerah
JAMKESMAS: Jaminan Kesehatan Masyarakat
JAMSOSTEK: Jaminan Sosial Tenaga Kerja
JKN: Jaminan Kesehatan Nasional
